# Improving the quality of nurse clinical documentation for chronic patients at primary care clinics: A multifaceted intervention

**DOI:** 10.4102/curationis.v38i1.1497

**Published:** 2015-09-25

**Authors:** Ozayr H. Mahomed, Salsohni Naidoo, Shaidah Asmall, Myra Taylor

**Affiliations:** 1 Discipline of Public Health Medicine, University of KwaZulu-Natal, South Africa; 2 National Department of Health, Civitas Building, South Africa

## Abstract

**Background:**

Deficiencies in record keeping practices have been reported at primary care level in the public health sector in South Africa. These deficiencies have the potential to negatively impact patient health outcomes as the break in information may hinder continuity of care. This disruption in information management has particular relevance for patients with chronic diseases.

**Objectives:**

The aim of this study was to establish if the implementation of a structured clinical record (SCR) as an adjunct tool to the algorithmic guidelines for chronic disease management improved the quality of clinical records at primary care level.

**Method:**

A quasi-experimental study (before and after study with a comparison group) was conducted across 30 primary health care clinics (PHCs) located in three districts in South Africa. Twenty PHCs that received the intervention were selected as intervention clinics and 10 facilities were selected as comparison facilities. The lot quality assurance sampling (LQAS) method was used to determine the number of records required to be reviewed per diagnostic condition per facility.

**Results:**

There was a a statistically significant increase in the percentage of clinical records achieving compliance to the minimum criteria from the baseline to six months post-intervention for both HIV patients on antiretroviral treatment and patients with non-communicable diseases (hypertension and diabetes).

**Conclusions:**

A multifaceted intervention using a SCR to supplement the educational outreach component (PC 101 training) has demonstrated the potential for improving the quality of clinical records for patients with chronic diseases at primary care clinics in South Africa.

## Introduction

South Africa is experiencing a ‘profound health transition’ (Mayosi *et al.* 2009:1) characterised by four simultaneous epidemics (Bradshaw *et al.* 2003). This quadruple burden of disease consists of the Human Immune Deficiency Virus (HIV) that manifests as Acquired Immune Deficiency Syndrome (AIDS), accidental and non-accidental injuries, other communicable diseases such as tuberculosis, diarrhoea and pneumonia, which interact in vicious negative feedback loops with malnutrition and the growing incidence of non-communicable diseases (NCDs) (Norman *et al.* 2006).

NCDs were the highest contributor to the mortality (40.8%) and to the disability adjusted life-years (DALYs) (33%) and the third most significant contributor to years of life lost (YLL) (22.8%) in 2000 (Norman *et al.* 2006). The World Health Organisation (WHO) estimated that NCDs were responsible for 28% of the total burden of disease measured by DALYs in South Africa in 2004. These estimates indicate that the burden from non-communicable diseases in South Africa is two to three times higher than in developed countries, and is similar to countries within the highest quintile for NCDs (WHO 2004).

South Africa has the largest number of citizens living with HIV in the world with approximately 6.4 million in 2012 (Government of South Africa 2014) and the largest antiretroviral treatment (ART) programme in the world with approximately 2.7 million patients receiving ART in 2014 (Government of South Africa 2014). The unprecedented roll-out of ART has transformed AIDS into a chronic disease, as people with it are living longer and ageing, and are developing non-AIDS related chronic diseases similar to the rest of the population. Some NCDs are related to HIV infection itself and to the side-effects of some of the medicines used to treat HIV infection (Johnson 2012).

As the burden of chronic diseases (both communicable and NCDs) increases, providing affordable and effective care to the often large and increasing numbers of people will be an immense challenge for the health system.

The successful management of chronic diseases requires coordination of services for individuals over an extended period (Rabkina & El-Sadra 2011). A patient's medical record (PMR) is an essential tool to ensure coordination and continuity of care (Medical Protection Society 2014). The main purpose of the medical record is to record the facts about the patient's health during the current attendance at the health care facility, and subsequently for the continuing care of the patient when they require health care in the future (WHO 2002). In addition, medical records are a medico-legal resource and are used in the production of health care statistics, for clinical audits, medical research, and performance monitoring of staff and services (Mann & Williams 2003).

## Problem statement

Anecdotal evidence about the quality of record keeping provided by the operational managers of the PHCs in South Africa support those of other studies conducted in developing countries that indicate there were deficiencies in record keeping practices such as duplication, incomplete data, and data inaccuracies. This has particular relevance for patients with chronic diseases who interact with the health service over several years as such deficiencies in record keeping negatively impact on patient management hindering continuity of care.

## Background

The preceding 10 years (2004−2013) have seen a concerted effort directed at addressing the HIV and AIDS epidemic in South Africa (National Department of Health [NDOH] 2012). The HIV programme has developed many innovative approaches to the comprehensive management of patients that include task shifting and sharing, adherence counselling and support, defaulter tracing initiatives, the use of multidisciplinary teams and community engagement. In addition, SCRs and registers for monitoring and evaluation have been implemented, mentoring and support for improving quality of care, appropriate referral mechanisms and appropriate laboratory testing have served to strengthen and facilitate the unprecedented roll-out of ART (Joint United Nation Programme on HIV and AIDS 2011).

However, the interventions directed at the HIV programme were delivered using a vertical approach and other programmes including NCDs and mental health, did not receive the same attention. Since 2009, the NDOH has placed a renewed focus on strengthening the management of chronic diseases to increase life expectancy and strengthen the health system's effectiveness (NDOH 2010a). The proposed strategies to target chronic diseases include re-organising and improving the functioning of clinical services with the extension of care of both communicable and non-communicable into communities. This is being implemented through an integrated chronic disease management (ICDM) framework which is being implemented through the re-engineered PHC framework (NDOH 2010b).

**FIGURE 1:**
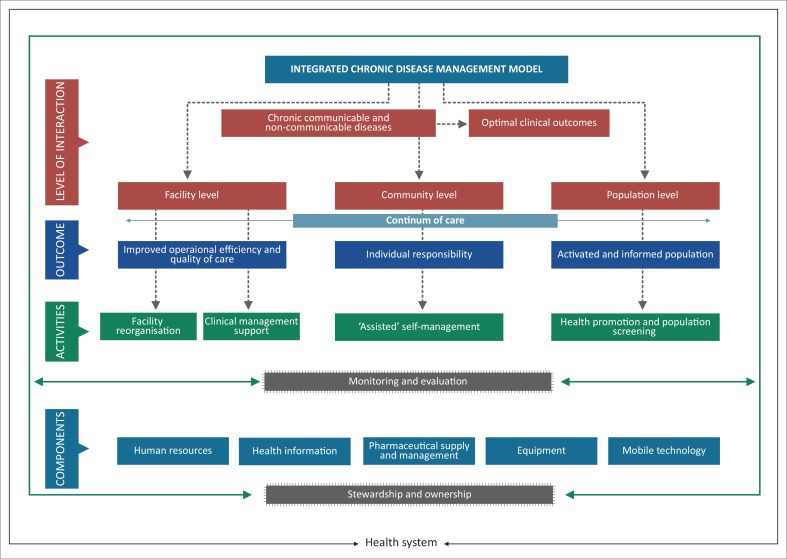
Integrated chronic disease management model.

Leveraging on the innovations of the HIV programme and utilising the primary health care re-engineering framework, the ICDM model has been introduced as a vehicle to improve the management of chronic diseases (Asmall & Mahomed 2013) (Figure 1).

The ICDM represents a fundamental shift in the management of patients with long term chronic diseases evolving from a ‘disease-centred model’ towards a ‘patient centred care’ approach (International Alliance of Patients’ Organizations 2007:10), where patients are informed, educated and participate in their own care. As a result of this shift in focus in patient management, a patient attending the PHC facility can potentially be treated by a number of health care providers during any period of care. It is essential that there is proper hand-over and communication between and amongst the various health care service providers. In order to facilitate this the PMR is required to provide a clear depiction of the patients care to date.

The ICDM consists of four inter-related intervention phases, namely, facility re-organisation, clinical supportive management, assisted self-supportive management, and strengthening of support systems and structures outside the facility that interact at the health service level, individual patient level and the community and population level to ensure a seamless transition to assisted self-management within the community (Figure 1).

The clinical supportive management phase of the ICDM includes training in and implementation of Primary Care 101 (PC101) Clinical Guidelines (NDOH 2011/2012)as a standardised approach to clinical management, and the application of a SCR for patients with chronic diseases that supports the appropriate management of patients according to the pre-defined algorithms.

The aim of this study was to establish if the implementation of a SCR as an adjunct tool to the algorithmic guidelines for chronic disease management improved the quality of clinical records at primary care level.

### Study objectives

The objectives of the study were to determine the quality of clinical records pre-intervention and to assess whether there were any changes in the quality of clinical records at six months post-intervention

### Literature review

The successful management of chronic diseases requires coordination of services for individuals over an extended period (Rabkina & El-Sadra 2011). Despite their importance, proper and good quality medical recording have not been prioritised adequately. A study conducted at a surgical department at a district hospital in KwaZulu-Natal showed numerous deficiencies in surgical notes, particularly with recording of patients’ appropriate history, acknowledgment and interpretation of radiological and laboratory requests and legibility (Chamisa & Zulu 2007).

In an effort to improve the quality and safety of clinical care, increasing patient expectations and the increased instances of medical litigation, the need for clinical coding as well as the transition to electronic medical records a renewed focus has been placed on the structure and content of the clinical record (Pullen & Loudon 2006).

The implementation of a structured clinical record has shown overall improvement in the quality of clinical documentation. Pro forma clinical records introduced at a South African regional level hospital in 2012 demonstrated an overall improvement in documentation. Of significance, there was an improvement in the documentation of maternal HIV status, children HIV status and children TB risk assessment (all *P* < 0.001). This study supports the assertion that tailor-made pro forma documentation may have an important role to play in improving record keeping in low-resource settings (Goenka *et al.* 2014)

This was further supported by a study that was conducted at Charlotte Maxeke Johannesburg Academic Hospital (CMJAH) to determine the impact of a new structured record form on the quality of patient records of emergency department (ED) records taken by doctors. This showed a significant improvement from baseline to both one month (*p* < 0.05) and three months (*p* < 0.001) after the introduction of the structured form. The difference between results at one and three months was not significant (*p* > 0.05). At baseline, only 6 of the 16 variables included in the structured form were recorded in 90% of the records, whilst at three months, 13 of 165 were captured more than 90% of the time. Levels of the legibility of records reached more than 90% even at baseline (Motara* et al.* 2013).

Although professional nurses form the backbone of the healthcare delivery system, there is, however, a paucity of literature that has focussed on the quality of nursing records at primary care clinic level.

## Research method and design

### Context

The study was conducted across the following three ICDM initiating districts: Dr Kenneth Kaunda district (DKK), North-West province, West Rand Health district (WRH), Gauteng province, and the Bushbuckridge (BBR) sub-district within the Ehlanzeni district, Mpumalanga province.

DKK district has a population of approximately 807 000. Health services are delivered by 1 regional and 3 district hospitals, 9 community health centres, 27 clinics, 6 satellite clinics and 2 mobile health service units.

The WRH district has a population of approximately 900 000. The West Rand district municipality has a total of 60 health facilities comprising 1 regional hospital, 2 district hospitals, 4 community health care centres and 39 PHC clinics.

BBR sub-district is a presidential nodal point with an estimated population of 509 967. Three district hospitals, 2 community health care centres, 36 operational clinics and 5 mobile clinics form the platform to deliver health services.

### Intervention

Primary Care 101 (PC 101) is a 101-page clinical guideline which covers the management of all common symptoms and diseases seen in adolescents and adults who seek care from PHC services (NDOH 2011/2012). All clinical staff at the PHC facility are trained to use PC 101 through onsite educational outreach, delivered over a prolonged period (8−12 weeks). Regular short training sessions are conducted by facility trainers who are nurses drawn from the PHC facility.

A structured clinical record (SCR), which incorporated the PC 101 and national guidelines for the management of patients with chronic diseases, was designed by the principal investigator and senior technical advisor from the NDOH. The SCR was implemented one month after the baseline assessment of all the clinics and prior to all professional nurses receiving PC 101 training. The SCR comprises a double-sided, A4-page which is inserted into each patient's folder. On the first side data for the first six months since patient enrolment on the chronic disease programme are collected and on the second data for the second six months are documented (Figures 2a and (2b). The SCR consists of different sections required for a complete PMR. The first section requires the health care worker to complete the administrative details of the patient and a tick box for noting the chronic disease for which the patient is being treated. The second section provides for the entry of routine vital signs measured at each patient visit including blood pressure, random blood sugar, patient's weight, height, body mass index, urine dipstick results and pulse measurements. The history section has categorical variables on symptoms of acute exacerbations, limitation of activity, hospitalisation or doctor visits, medication adherence and side-effects and the use of tobacco, alcohol or illicit drugs. The examination section of the form requires recording of positive or negative findings of the respiratory, cardiovascular, abdominal, neurological, mental status and peripheral vascular examination in order to detect early signs of complications. The medication section requires entry of the prescribed medication. This is followed by a section that allows the service provider to indicate the health education and health promotion provided to the patient.

**FIGURE 2a:**
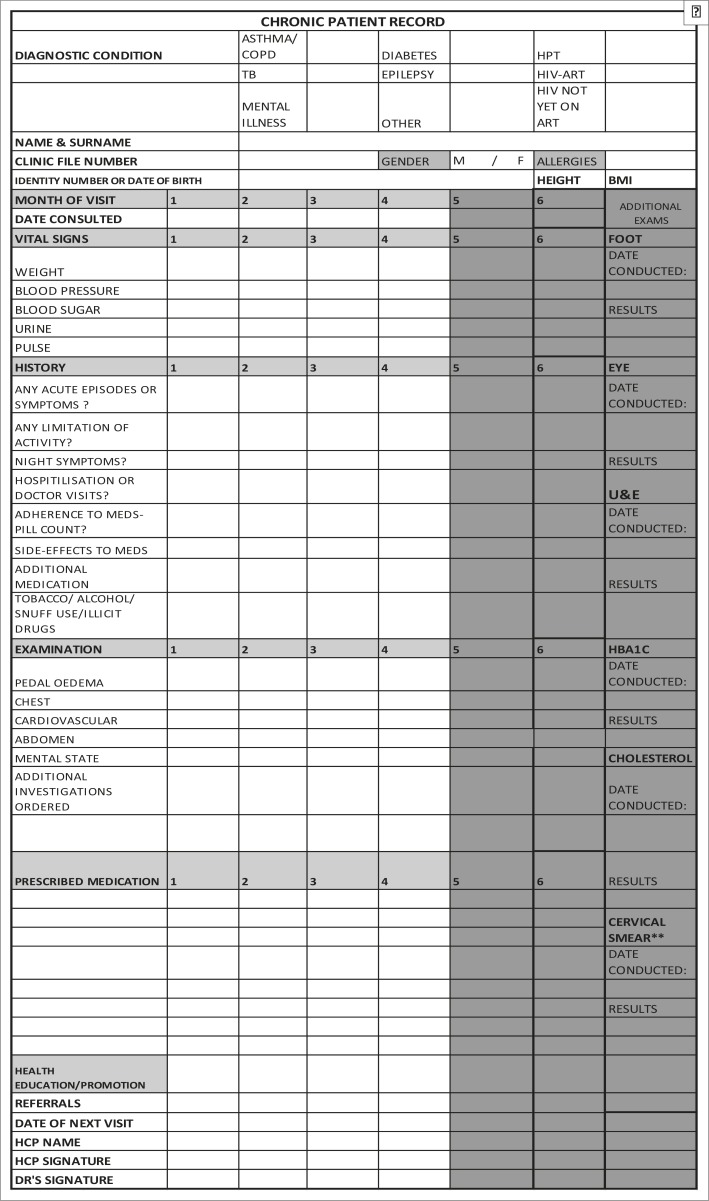
Chronic patient records: month one to six.

**FIGURE 2b:**
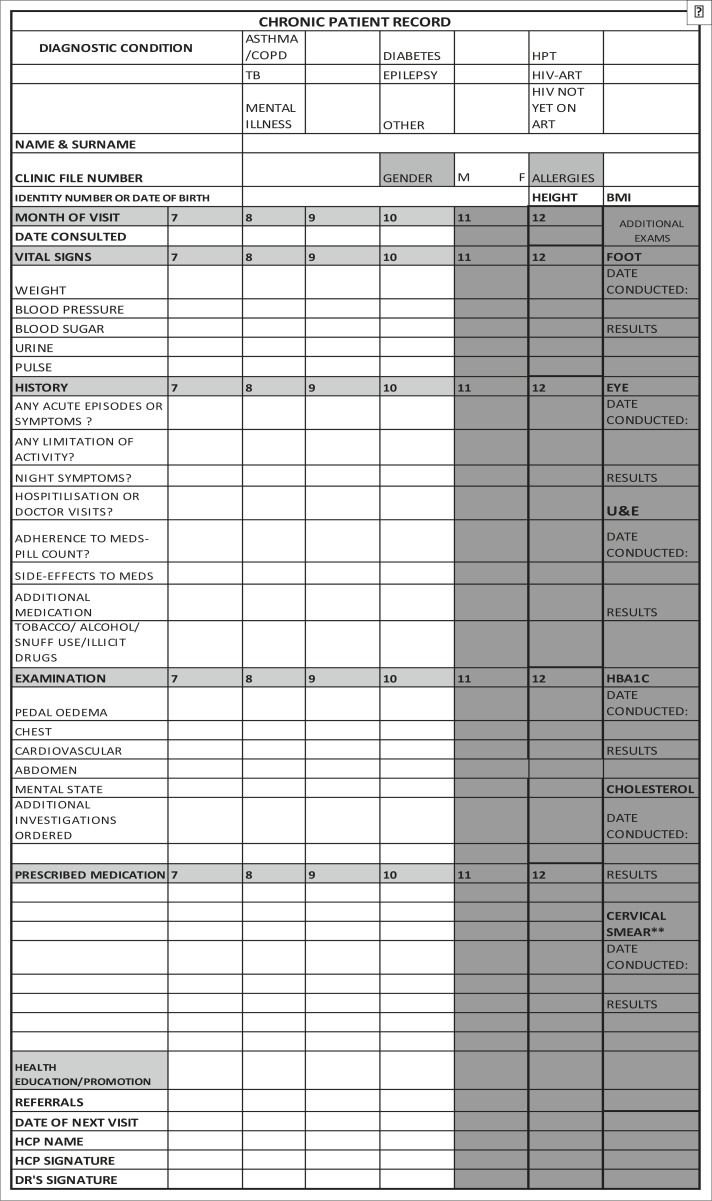
Chronic patient record: Months 6–12.

The last section requires the service provider to record his or her name and sign the record. A separate column is provided for the recording of relevant foot and eye examinations, biochemistry test results such as urea and electrolytes, glycosylated haemoglobin and total cholesterol levels, and cervical smear results annually or six-monthly as per the defined protocol.

The principal investigator and senior technical advisor conducted onsite training for all clinical and administrative staff at all the ICDM initiating PHC facilities on the correct application of the SCR. A standard operating procedure was made available to the ICDM initiating facilities and was included in the ICDM implementing guide (Asmall & Mahomed 2013).

The control group did not receive the PC 101 guidelines or training and the SCR was not implemented at the control facilities.

### Study design

This study falls within the scope of implementation research and could be considered as a pragmatic trial (Zwarenstein *et al.* 2008). A quasi-experimental before and after study with a comparison group was conducted between May 2012 and January 2013.

### Study population and sampling strategy

The ICDM was initiated at 42 PHC facilities across three districts in South Africa. Of these, 20 PHC clinics (6 facilities in DKK, 8 in WRH and 6 in BBR) were selected as intervention sites whilst 10 PHC clinics (3 in DKK, 4 in WRH and 3 in BBR) were selected as comparison sites.

A multi-stage stratified random sampling strategy was utilised in selecting the intervention clinics for the evaluation. The clinics were stratified to ensure representation from all sub-districts (*n* = 12) and then stratified according to chronic disease patient case load to ensure representation from the facilities with high, medium and low chronic patient case load. The facilities were then randomly selected from each strata using the random number table.

The comparison clinics were conveniently selected by the district PHC team. A request was made for matching facilities in terms of catchment population, chronic patient load to the ICDM clinics and representation from each sub-district; however, this was not strictly adhered to.

### Sample size

The Lot Quality Assurance sampling (LQAS) method was used. Each PHC facility represented a single lot. The number of records required to be reviewed per chronic disease per facility was calculated using a confidence level of 95%, anticipated population prevalence of 30%, and standard of 80%, the maximum level of sampled population showing the characteristic (*d*) of 2 and a population size greater than 100. This required a sample size of 19 records be extracted to represent a single lot (Lwanga & Lemeshow 1991).

## Selection of sample

The chronic disease patient register and the ART patient register were used as the sampling frame for the study. Patients who attended the intervention facilities over the last six months were sampled randomly. The random number table was used to generate the number and the corresponding patient record.

### Data collection

The data were collected at baseline (May 2012) prior to implementation of the training, at three months (October 2012) and six months (January 2013) post facility based PC 101 training and SCR implementation. Data were collected through a review of the clinical records for patients attending the facility in the previous three months for the following three chronic diseases: patients on ART, diabetes and hypertension. The data collection team consisted of skilled professional nurses with clinical and research experience. A structured data collection tool was designed for the review of the patient clinical record. The variables that were measured are described later in the article.

The data collection tool was piloted at two clinics in KwaZulu-Natal and the adjustments were made to accurately reflect the content of the clinical records. The principal investigators conducted site visits and reviewed records at 20% (*n* = 6/30) of the selected clinics to assure the quality of the record review process.

### Data analysis

The patient record reviews were analysed against clinical criteria listed in Table 1.

**TABLE 1:** Criteria for assessing medical records.

Criteria for measurement	Hypertension	Diabetes	Patient on art
Date that the patient commenced art recorded			x
Evidence of appropriate history taking in terms of symptoms experienced	x	x	x
Recording of who clinical staging			x
Body weight recorded at each visit	x	x	x
Blood sugar- random recorded at each visit for diabetes but once annually for hypertensive	x	x	
Body mass index recorded (once annually)	x	x	x
Urine dipstick at least once in 6 months if normal	x	x	x
Baseline and annual u& e conducted and results recorded	x	x	x
hba1c conducted and recorded at least once annually		x	
Annual opthalmic screening examination recorded		x	
Comprehensive foot examination conducted and recorded annually		x	
Medication and dosage recorded at each visit	x	x	x
Evidence of health promotion- diet, exercise, smoking cessation, weight reduction and alcohol reduction noted	x	x	x
Change in medication or increase in dosage of medication noted	x	x	x
History of patient missing medication or pill count noted?	x	x	x
Any side-effects noted and graded	x	x	x
Baseline and 6 month CD4 count recorded			x
Baseline and annual viral load level recorded			x
Baseline HB collected and results recorded:			x
Baseline safety alt collected and results recorded:			x
TB screening recorded at every visit			x
IPT prophylaxis recorded			x
Cotrimoxazole prophylaxis recorded			x

A positive response for each criterion was accorded a score of 1. All positive responses per record were summed to obtain the total for each patient record. During the baseline, we calculated the median score for each condition in terms of compliance to the measurement criteria. This median score (7 for diabetes and hypertension and 11 for patients on ART) was used as a benchmark for an acceptable quality record. Each facility was then evaluated to determine the number of clinical records that met or exceeded the benchmark.

The total number of all records that scored above the benchmark was then summed and divided by the sample size to obtain the mean coverage for the intervention and control sites for each chronic diagnosis. A paired *t*-test with unequal sample variance was used to compare the average district coverage per chronic disease at baseline (prior to the intervention), and at three months from baseline, and six months, post PC 101 training. A 95% confidence interval was reported with the accepted level of significance being 0.05 (α = 0.05).

## Ethical consideration

This study received ethical approval from the Biomedical Research Ethics Committee at the University of KwaZulu-Natal (UKZN) (BE 006/12). Permission to undertake the study was obtained from the national, provincial and district Departments of Health including the facility managers. All data were collected anonymously.

## Results

### Baseline assessment

The records for patients with HIV on ART achieved the highest coverage in terms of compliance with assessment criteria at both the intervention and comparison sites. Sixty five percent (74/114) in DKK, 44% (67/152) in WRH and 40% (46/114) in BBR of the records for HIV positive patients on ART that were reviewed at the intervention sites during the baseline assessment, complied with 11 or more criteria. Although the comparison sites in WRH achieved a higher compliance (67% of records [51/76]) for HIV positive patients on ART than the intervention sites in WRH, this was not statistically significant (*p* > 0.05).

There was a statistically significant difference (*p* < 0.01) in the percentage of records achieving compliance with seven or more criteria for hypertension records, between the intervention sites in DKK compared to both the intervention sites in WRH and BBR and between the comparison sites in DKK and BBR (*p* < 0.05). Sixty one percent (69/114) in DKK, 22% (33/152) in WRH and only 3% (3/114) of records for patients with hypertension that were reviewed at the intervention sites achieved the benchmark of seven or more criteria. The comparison sites in DKK achieved a higher baseline coverage with 58% (33/57) of the records complying with seven or more criteria.

Seventy five percent in DKK (43/57) and 53% in WRH (40/76) of clinical records for diabetic patients at the comparison sites in DKK and WRH complied with seven or more criteria. Fifty six percent (64/114) in DKK, 29% (31/152) in WRH and only 3% in BBR (3/114) of records for patients with diabetes, at the intervention sites achieved the benchmark of seven or more criteria. There was a significant difference (*p* < 0.05) between the intervention sites in DKK compared to WRH and BBR (Table 2).

**TABLE 2:** Baseline compliance of clinical records with assessment measures in the three districts (May 2012).

Mean coverage	Dr Kenneth Kaunda district	Dr Kenneth Kaunda district (comparison)	West Rand Health district	West Rand Health district (comparison)	Bushbuckridge subdistrict	Bushbuckridge subdistrict (comparison)
Patients on ART	65% (95% CI: 58% – 71%)	56% (95% CI: 49% – 63%)	44.1% (95% CI: 39.1% – 49.0%)	67.1% (95% CI: 59.6% – 74.7%)	40.4% (95% CI: |33.8% – 46.9%)	31.6% (95% CI: 18.9% – 44.2%)
Number of facilities below standard:	2	1	5	2	4	2
Hypertension	60.5% (95% CI: 57.3% – 63.7%)	57.9% (95% CI: 51.8% – 64.0%)	22% (95% CI: 19% – 25%)*	18% (95% CI: 13% – 24%)	3% (95% CI: 2% – 3%)	4% (95% CI: 1% – 6%)
Number of facilities below standards	1	1	7	3	6	3
Diabetes	56% (95% CI: 52% – 61%)	75% (95% CI: 69% – 82%)	20% (95% CI: 18% –23%)	53% (95% CI: 49% – 56%)	3% (95% CI: 2% – 3 %)	4% (95% CI: 1% – 6%)
Number of facilities below standards	3	0	7	1	6	3

ART, antiretroviral treatment.

### Post-intervention in Dr Kenneth Kaunda district

There was a statistically significant (*p* < 0.05) increase in the percentage of clinical records for patients with hypertension (61% − 90%) and diabetes (56% − 73%) that achieved the benchmark of a record seven criteria at the intervention sites, three months post SCR implementation intervention (October 2012). Although the percentage of clinical records for HIV positive patients on ART that achieved the benchmark of 11 criteria, increased by 11% (65% − 76%) this was not statistically significant (*p* > 0.05). Whilst not statistically significant the quality of records at the control sites in DKK three months post SCR implementation also improved with 96% of clinical records for HIV positive patients on ART, 96% of clinical records for hypertension, and 96% of clinical records for diabetes records meeting the benchmark.

At the six months post SCR implementation assessment (January 2013), there was a further increase in the percentage of clinical records from the three months post SCR implementation assessment (October 2012) for hypertension (90% − 96% at the intervention sites and 96% − 100% at the control sites) that achieved the benchmark. However, between three months (October 2012) and six months (January 2013) post SCR implementation there was a decline in the percentage of clinical records for HIV positive patients on ART (76% − 73% at the intervention sites and 96% − 67% at the control sites) and diabetes (90% − 89% at the intervention sites and 96% − 88% at the control sites) that achieved the benchmark (Table 3).

**TABLE 3:** Pre-intervention three months and six months post-intervention mean compliance scores in Dr Kenneth Kaunda district.

Mean coverage	Average coverage intervention sites	Average comparison control sites	Number of facilities performing below standard- intervention sites	Number of facilities performing below standard- control sites
**HIV positive patients on ART**				
Pre-PC 101 training	64.9% (95% CI: 58.5% – 71.4%)	56.1% (95% CI: 48.8% – 63.4%)	2	1
3 month post PC 101 training	76.3% (95% CI: 71.1% – 81.5%)	96.5% (95% CI: 94.5% – 98.5%)	2	0
6 month post PC 101 training	72.8% (95% CI: 69.5% – 76.2%)	66.7% (95% CI: 59.5% – 73.8%)	1	1
**Hypertension**				
Pre-PC 101 training	60.5% (95% CI: 57.3% – 63.7%)	57.9% (95% CI: 51.8% – 64.0%)	1	1
3 month post PC 101 training	90.4% (95% CI: 87.8% – 92.9%)	96.5% (95% CI: 94.5% – 98.5%)	0	0
6 month post PC 101 training	95.6% (95% CI: 94.4% – 96.8%)	100% (95% CI: 100% – 100%)	0	0
**Diabetes**				
Pre-PC 101 training	56.14% (95% CI: 51.7% – 60.5%)	75.4% (95% CI: 69.2% – 81.6%)	3	0
3 month post PC 101 training	90.4% (95% CI: 87.8% – 92.9%)	96.5% (95% CI: 94.5% – 98.5%)	2	0
6 month post PC 101 training	89.5% (95% CI: 85.6% – 93.4%)	87.7% (95% CI: 82.1% – 93.4%)	1	1

ART, antiretroviral treatment.

### West Rand Health district

There was a statistically significant (*p* < 0.01) increase in the percentage of clinical records for HIV positive patients on ART (44% − 92%), patients with hypertension (22% − 88%) and diabetes (20% − 88%) that achieved the benchmark of 11 and 7 criteria respectively at the intervention sites, three months post-intervention (October 2012). Although not statistically significant the proportion of records that achieved the benchmark for an acceptable quality of record at the control sites in WRH three months post SCR implementation improved for HIV positive patients on ART but declined statistically significantly (*p* < 0.01) for clinical records for hypertension from 18% − 5% and for clinical records for patients with diabetes (53% − 29%).

There was a statistically significant (*p* < 0.01) increase from baseline to six months post-intervention for the quality of clinical records for patients with hypertension and diabetes (Table 3). However, the percentage of clinical records that achieved the benchmark of a record of an acceptable quality declined from the three month (October 2012) to six month assessment (January 2013) for patients with HIV on ART (92% − 65%), for patients with hypertension (88% − 71%) and patients with diabetes (88% − 84%) (Table 4).

**TABLE 4:** Pre-intervention, three months and six months post-intervention mean compliance scores in West Rand Health district.

Mean coverage	Average coverage intervention sites	Average coverage control sites	Number of facilities performing below standard- intervention sites	Number of facilities performing below standard- control sites
**HIV positive patients on ART**				
Pre-PC 101 training	44.1% (95% CI: 39.1% – 49.0%)	67.1% (95% CI: 59.6% – 74.7%)	5	2
3 month post PC 101 training	92.1% (95% CI: 89.8% – 94.4%)	69.7% (95% CI: 60.7% – 78.8%)	1	1
6 month post PC 101 training	66.4% (95% CI: 62.3% – 70.6%)	64.5% (95% CI: 54.0% – 74.9%)	3	1
**Hypertension**				
Pre-PC 101 training	21.7% (95% CI: 18.9%–24.5%)	18.4% (95% CI: 12.7% – 24.2%)	7	3
3 month post PC 101 training	88.2% (95% CI: 84% – 92.3%)	5.3% (95% CI: 3.4% – 7.1%)	1	4
6 month post PC 101 training	71.1% (95% CI: 66.7% – 75.4%)	26.3% (95% CI: 14% – 38.6%	2	3
**Diabetes**				
Pre-PC 101 training	20.4% (95% CI: 18.1% – 22.7%)	52.6% (95% CI: 49.4% – 55.9%)	7	1
3 month post PC 101 training	88.2% (95% CI: 84.2% – 92.1%)	28.95% (95% CI: 10% – 18.95%)	1	4
6 month post PC 101 training	83.55% (95% CI: 79.3–87.5% – 75%)	46.05% (95% CI: 35.3% – 56.8%	1	3

ART, antiretroviral treatment.

### Bushbuckridge sub-district (BBR)

At three months post SCR implementation, only the patient records for HIV positive patients showed an increase from 41% − 51% at the intervention sites and from 32% − 53% at the control sites in the percentage of clinical records that achieved the benchmark. At six months post SCR, there was a statistically significant (*p* < 0.01) increase in the proportion of clinical records for patients with hypertension (3% to 91%) at the intervention sites and (4% − 84%) at the control sites, and patients with diabetes (3% − 91%) at the intervention sites and (4% − 84%) at the control sites that achieved the benchmark of seven criteria. Although the percentage of clinical records for HIV positive patients which achieved the benchmark of 11 criteria increased from 41% − 82% at the intervention sites and 32% − 90% at the control sites between the baseline and six months post-intervention this was not statistically significant (Table 5). However, of particular note is the deterioration in the percentage of clinical records for diabetes and hypertension that achieved the minimum level of compliance at three months.

**TABLE 5:** Pre-intervention, three months and six months post-intervention mean compliance scores in Bushbuckridge subdistrict.

Mean coverage	Average coverage intervention sites	Average comparison sites	Number of facilities performing below standard- intervention sites	Number of facilities performing below standard- comparison sites
**HIV positive patients on ART**				
Pre-PC 101 training	40.4% (95% CI: 33.8% – 46.9%)	31.6% (95% CI: 18.9% – 44.2%)	4	2
3 month post PC 101 training	50.9% (95% CI: 42.5% – 59.2%)	52.6% (95% CI: 35.9% – 69.4%)	3	2
6 month post PC 101 training	81.6% (95% CI: 76.5% – 86.6%)	89.5% (95% CI: 84.8% – 94.1%)	1	0
Hypertension				
Pre-PC 101 training	2.6% (95% CI: 2.2% – 3.1%)	3.5% (95% CI: 1.5% – 5.5%)	6	3
3 month post PC 101 training	0	0	6	5
6 month post PC 101 training	91.2% (95% CI: 89.3% – 93.2%)	84.2%(95% CI: 78.9% – 89.5%	1	1
**Diabetes**				
Pre-PC 101 training	4.4% (95% CI: 3.4% – 5.4%)	7.02% (95% CI: 6.0% – 8.03%)	6	3
3 month post PC 101 training	0	0	6	5
6 month post PC 101 training	72.81% (95% CI: 67.9% – 77.7%)	63.2% (95% CI: 54.04 – 72.7%)	1	1

ART, antiretroviral treatment.

## Discussion

The *Guidelines for Good Record Keeping* published by the Health Professional Council of South Africa identified bio-psychosocial history of the patient, assessment of the patient's condition, proposed clinical management of the patient, the medication and dosage prescribed including test results, amongst the minimum criteria for clinical records (Health Professional Council of South Africa 2008). Although these are minimum criteria and there should be 100% compliance, the evidence obtained from previous studies indicates deficiencies in medical record keeping (Chamisa & Zulu 2007). The SCR was designed to achieve compliance with the above criteria as well as promote continuity of care.

The results of the study demonstrate a statistically significant increase in the proportion of clinical records achieving compliance with the minimum criteria from the baseline to six months post-intervention for both HIV patients on ART and patients with NCDs (hypertension and diabetes). The minimum criteria were calculated based on the median level of compliance with assessed criteria at the baseline assessment. This is because of the paucity of studies or audits conducted on medical records in the primary care setting in South Africa.

At the baseline (pre-intervention), the clinical records for HIV patients on ART had the highest mean coverage in terms of compliance to assessed criteria across all three districts. This is reflective of the intense focus on HIV and AIDS with the introduction of national ART stationery and numerous training initiatives for professional nurses such as ’Nurse initiated management of antiretroviral treatment’ (NIMART) (Georgeu *et al.* 2012), ‘Practical approach to lung health in South Africa’ (PALSA) (Bheekie *et al.* 2006) and ‘PALSA-PLUS’ (Stein *et al.* 2008).

An important finding of this study is the statistically significant improvement in the mean coverage in terms of compliance with assessed criteria across all three districts in the clinical records for patients with hypertension and diabetes. This is in contrast to a previous study conducted in public sector community health centres (CHCs) in Cape Town in 1999 and 2000 that failed to demonstrate any benefit of using a SCR which incorporated variables of the national guidelines for the management of type 2 diabetes and hypertension (Steyn *et al*. 2013).

The improvement could be attributed to the ease with which the health care professionals adopted the SCR at facility level and across all three districts. This is in part as a result of the simplicity of the SCR and secondly the appropriateness of the design that was facilitated by consulting district family physicians, district clinical programme managers and facility operational managers who provided input into the development of the SCR.

A major contributing factor to the uptake and application of the SCR was its availability. The printing of the SCR, PC 101 training, including accommodation of staff during the master training was funded by the external support partner with no printing or logistical costs that needed to be covered by the province or district. This has important implications as the success of this initiative is dependent on identifying sources of funds for the printing of the SCR, further training, logistics for the delivery of the SCR to facilities and providing administrative support to the various facilities (Docimo *et al*. 2000). The lag in improvement in BBR is attributed to the poor administrative and logistical arrangements in ensuring availability of the SCR to facilities.

Of concern is the decline in the mean compliance scores of the clinical records for HIV patients on ART in DKK at the intervention sites and for all chronic diseases (HIV patients on ART, hypertension and diabetes) in WRH between three and six months post SCR implementation. This decline could be attributed to the rotation of staff across the services. This is supported by a study that showed the re-allocation of clinical staff away from clinical areas may negatively affect the ability to track changes, and that any intervention aimed at changing service provision must take into consideration staff rotation to be successful in changing practice over the long term (English *et al.* 2009).This has important implications for the scale-up of the implementation of the SCR as well as for improved quality of clinical records.

An interesting finding of this study is the improvement in the mean compliance scores of the clinical records at the comparison sites between the baseline assessment and six months post SCR implementation assessment. A potential reason for underpinning this improvement is the lateral linkages between facilities that allowed for sharing of their experiences and best practices. Furthermore, many of the operational managers were intrinsically motivated to improve the quality of clinical records at their facilities and utilised the assessment tool as a template for training the staff on the critical elements of a complete clinical record. Although, these improvements at the comparison sites were not anticipated, the results provide confidence for the successful uptake of the SCR because of the prevailing supporting culture willingness to change and staff cooperation that are critical facilitators for uptake of quality improvement interventions (Wolfson *et al*. 2009)

The focus of this study was on the global clinical record rather than individual process measures, although the individual measures were evaluated for compliance. The district was considered as the intervention unit. This facilitated the application of the LQAS methodology. LQAS has been identified as an improved method of sampling in surveys to measure health service coverage, but also to assess the performance in supervision areas or lots, which together make up the overall district or project area (Valadez *et al*. 2001). This methodology does not seek to obtain precise estimates, but aims to facilitate the decision making process regarding the quality levels of the indicators that are being examined (Corbella & Grima 1999). This allows the district to target interventions towards underperforming supervision areas.

### Study limitations

Although due diligence was exercised in maintaining the scientific integrity of the study, the inherent design and nature of the study predisposed it to limitations. The study was conducted in a real environment rather than an experimental one. This resulted in the diffusion between the intervention and comparison sites.

The study was dependent on a sampling frame that was obtained from the disease specific registers. It is uncertain whether the cases recorded within the registers represent all the patients consulted at the facilities and therefore poses a selection bias risk.

Because of the small sample size, the confidence interval for LQAS is wider than other cluster sampling methods (Deitchler *et al*. 2007) reducing its precision.

## Conclusion and recommendation

A multifaceted intervention using a SCR to supplement the educational outreach component of the ICDM has demonstrated the potential for improving the quality of clinical records for patients with long term chronic illness. This has an important implication for continuity of care and optimal management of patients with chronic diseases (communicable and NCDs) in an environment of an ever increasing burden of chronic diseases.

In order for the SCR to be utilised and the achievable benefits sustained, it is important that on site capacity building on its correct usage is conducted across the facilities. Continuous supportive supervision needs to be provided by the PHC supervisors to monitor the quality of clinical records. In addition, a concerted effort should be made to ensure sufficient supply and distribution of the SCR form as its potential benefits may be compromised if the onus is left to the facilities to print this stationery. The SCR form should be implemented and tested in other settings prior to a full roll-out throughout the country.
